# Labor dystocia and oxytocin augmentation before or after six centimeters cervical dilatation, in nulliparous women with spontaneous labor, in relation to mode of birth

**DOI:** 10.1186/s12884-022-04710-2

**Published:** 2022-05-13

**Authors:** Cecilia Brüggemann, Sara Carlhäll, Hanna Grundström, Marie Blomberg

**Affiliations:** 1 Department of Obstetrics and Gynecology in Linköping, Department of Biomedical and Clinical Sciences, Linköping University, Linköping, Sweden; 2 Department of Obstetrics and Gynecology in Norrköping, Department of Biomedical and Clinical Sciences, Linköping University, Linköping, Sweden

**Keywords:** Oxytocin augmentation, Active labor, Labor dystocia, Cesarean section, Birth experience

## Abstract

**Background:**

The effects of diagnosing and treating labor dystocia with oxytocin infusion at different cervical dilatations have not been fully evaluated. Therefore, we aimed to examine whether cervical dilatation at diagnosis of dystocia and initiation of oxytocin infusion at different stages of cervical dilatation were associated with mode of birth, obstetric complications and women’s birthing experience.

**Methods:**

A retrospective cohort study, including 588 nulliparous term women with spontaneous onset of labor and dystocia requiring oxytocin augmentation. The study population was divided into three groups according to cervical dilatation at diagnosis of dystocia and initiation of oxytocin-infusion (≤ 5 cm, 6–10 cm, fully dilated) with mode of birth as the primary outcome. Secondary outcomes were obstetrical and neonatal complications and women´s experience of childbirth. Statistical comparison between groups using Chi-square and ANOVA was performed. The risk of operative birth (cesarean section and instrumental birth) was assessed using binary logistic regression with suitable adjustments (maternal age, body mass index and risk assessment on admission to the labor ward).

**Results:**

The cesarean section rate differed between the groups (*p* < 0.001); 12% in the ≤ 5 cm group, 6% in the 6–10 cm group and 0% in the fully dilated group. There was no increased risk for operative birth in the ≤ 5 cm group compared to the 6–10 cm group, adjusted OR 1.28 95%CI (0.78–2.08). The fully dilated group had a decreased risk of operative birth (adjusted OR 0.48 95%CI (0.27–0.85). The rate of a negative birthing experience was high in all groups (28.5%, 19% and 18%) but was only increased among women in the ≤ 5 cm group compared with the 6–10 cm group, adjusted OR 1.76 95%CI (1.05–2.95).

**Conclusions:**

Although no difference in the risk of operative birth was found between the ≤ 5 cm and 6-10 cm cervical dilatation-groups, the cesarean section rate was highest in women with dystocia requiring oxytocin augmentation at ≤ 5 cm cervical dilatation. This might indicate that oxytocin augmentation before 6 cm cervical dilatation could be contra-productive in preventing cesarean sections. Further, the increased risk of negative birth experience in the ≤ 5 cm group should be kept in mind to improve labor care.

**Supplementary Information:**

The online version contains supplementary material available at 10.1186/s12884-022-04710-2.

## Introduction

Labor dystocia is a common complication in nulliparous women and is strongly related to cesarean section (CS) and instrumental vaginal birth [1–3]. The majority of women diagnosed with labor dystocia receive oxytocin infusion to enhance uterine contractions [4, 5]. Currently, the definition of labor dystocia at different stages of labor is about to change. The American College of Obstetricians and Gynecologists (ACOG) [6] and the World Health Organization (WHO) [7, 8] suggests that active labor starts at 5 or 6 cm cervical dilatation, compared with the traditional definition by Friedman [9] stating 3–4 cm as the threshold for the start of active labor. Besides that active labor seems to start at a higher cervical dilatation than previously assumed [10, 11], the view of what is normal labor progress is debated and new cut offs for protracted labor at different cervical dilatations has been suggested [7].

The WHO and ACOG both changed their definitions of the start of active labor (recommendations) to stem the rising CS rates, as too much focus on cervical progression in early labor was viewed as a risk factor for CS and other interventions in labor that may negatively affect maternal and neonatal outcomes [6, 7]. Recommendations in Sweden still adhere to Friedman’s’ definition of active labor (4 cm cervical dilatation and expected cervical dilatation rate of 1 cm/hour from 4 cm with some slight moderations) [12], as do the Swedish recommendations for when to diagnose labor dystocia and initiate labor augmentation [5].

The CS rates in nulliparous women with a singleton term (≥ 37 + 0 gestational weeks) pregnancy, spontaneous onset of labor and vertex presentation, e.g. the Ten Group Classification System (TGCS) group 1 [13, 14] are relatively low in Sweden (range in 2020, 3–11%) compared with many other countries [15]. In Sweden, Region Östergötland has one of the lowest CS rates (5.9% in 2020) as an effect of an active improvement project, focusing on increasing the rate of spontaneous vaginal births in TGCS group 1 women. The TGCS group 1 was targeted for the improvement project as this relatively large group of women had much to gain by avoiding the first cesarean section. An increased vaginal birth rate in this group would reduce the risk of complications associated with CS both during the first birth and subsequent pregnancies and births [16].

It is still not clear whether diagnosing and treating labor dystocia with oxytocin before 5–6 cm cervical dilatation increases the risk of CS compared with after 5–6 cm [17]. Some studies, show decreased rate of CS performed due to labor dystocia by changing the recommendations of active labor [18] while others have not found the same compelling connection [19]. Most of these studies have been performed in countries with a generally high rate of CS [18, 19]. An important question that arises is whether the positive results in lowering the CS rate by changing the recommendations on labor dystocia from studies performed in a high CS rate context could be extrapolated to settings with relatively low numbers of CSs? A change in the definition of active labor and permitting a slower labor progress during active labor need to be thoroughly evaluated in relation to mode of birth and other outcomes in different contexts before the definition is fully implemented. Since the CS rate in Region Östergötland is low among women in the TGCS group 1, studying this group regarding degree of cervical dilatation at labor dystocia in relation to mode of birth would enable an evaluation in a new context (with low CS rate) compared with previous studies.

We hypothesized that a diagnose of labor dystocia and start of oxytocin infusion before 6 cm cervical dilatation increased the risk of cesarean section and instrumental birth, compared to labor dystocia diagnosed after 6 cm cervical dilatation in the TGCS group 1.

Thus, the primary aim of this study was to evaluate cervical dilatation at diagnose of labor dystocia and initiation of oxytocin infusion for labor augmentation in relation to mode of birth, in the TGCS group 1. Secondary outcomes were obstetrical and neonatal adverse outcomes and women´s experience of childbirth.

## Methods

### Study setting and participants

**Fig. 1:**
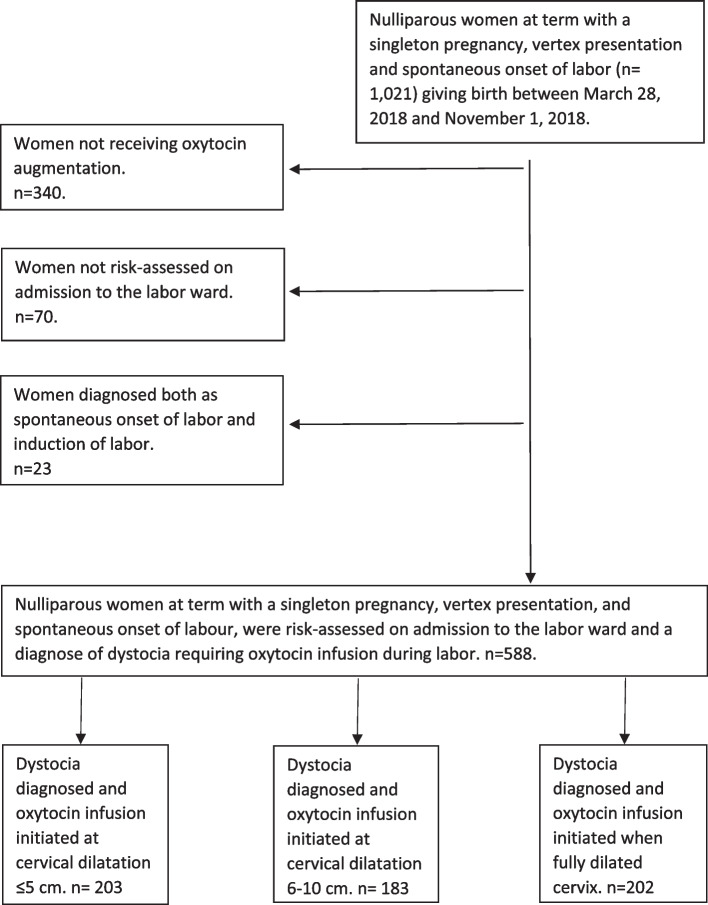
Flowchart of the study population

This retrospective cohort study included nulliparous women with a singleton and term (≥ 37 + 0 gestational weeks) pregnancy, spontaneous onset of labor and vertex presentation, TGCS group 1 [14], who gave birth from March to November 2018 at two hospitals in the Region Östergötland, which has approximately 5000 births per year combined. Further inclusion criteria were a documented risk classification on admission to the labor ward, a diagnosis of labor dystocia and initiation of oxytocin infusion in labor. A flowchart of the study population is presented in Fig. 1.

### Guidelines and recommendations

The participating hospitals followed the same national clinical guidelines concerning risk classification, active labor care, diagnosis of labor dystocia and oxytocin infusion initiation. Maternal and fetal risk classification was performed on admission to the labor ward using three risk categories: low, medium or high risk (Appendix 1). Active labor was defined as 4 cm of cervical dilatation, or one cm of cervical dilatation and a completely effaced cervix, painful, regular contractions and/or rupture of the membranes and progress of cervical dilatation within the following two hours, in accordance with the Swedish national recommendations [12]. Also in accordance with Swedish national guidelines, diagnosis of labor dystocia and initiation of oxytocin infusion was indicated when there was a delay in the expected cervical progress of one cm/hour for more than three hours, no progress in descent of the fetal head for one hour when fully dilated, or no progress after pushing actively for 30 min [5].

### Data collection and definition of variables

Maternal, obstetric and neonatal data were prospectively recorded in standardized electronic medical records (Obstetrix®) by the midwives and the physicians responsible for the care of the women. The maternal characteristics assessed were age and height, weight in early pregnancy, smoking during pregnancy, diabetes mellitus, hypertension, and asthma/lung disease. Maternal height and weight were measured at the first antenatal visit in gestational weeks 8–12, which enabled calculation of early pregnancy Body Mass Index (BMI, kg/m^2^). The obstetric characteristics that were extracted were: gestational age at birth, active labor time estimates, cervical dilatation at the start of oxytocin augmentation, epidural analgesia, mode of birth, occurrence of obstetric anal sphincter injury (OASI), postpartum hemorrhage (PPH) and women’s birthing experience according to the visual analog scale (VAS). At the postnatal ward, all women were asked by the midwife in charge at the postnatal ward to assess their overall birthing experience as a VAS-score ranging from 1 to 10, where 1 is a very negative experience and 10 is a very positive experience. This assessment of childbirth experience by VAS is a well-established routine in the postnatal care at the participating delivery units included in this study. A value of VAS 1–4 was considered a negative birthing experience according to the Swedish Pregnancy Registry [20].

Furthermore, the documented risk classification, assessed by the attending midwife on admission to the labor ward as low, medium or high (Appendix 1), was also extracted. The risk classification partly assesses the risk of labor dystocia by including parameters such as first trimester BMI ≥ 30, prolonged latent phase of labor, maternal psychological well-being, hypertensive disorders and preeclampsia, and fetal well-being (i.e., risk for infections in the newborn, intra uterine growth retardation, non-reassuring CTG (cardiotocography) and heavily meconium-stained waters). The neonatal variables assessed in the medical file were fetal birth weight and Apgar score < 7 at five minutes and umbilical cord arterial pH < 7.10. Data was extracted from the electronic medical records, except data concerning cervical dilation at diagnosis of labor dystocia and the risk classification on admission to the labor ward which were manually extracted from each medical record and added to the dataset. All variables were retrospectively extracted.

### Primary and secondary outcomes

The primary outcomes were mode of birth (spontaneous vaginal birth, instrumental vaginal birth, or CS) and a composite outcome of operative birth (CS and instrumental birth). Secondary outcomes were the use of epidural anesthesia, OASI grades III and IV, PPH > 1,000 mL, negative childbirth experience defined as VAS 1–4 and Apgar score < 7 at five minutes and cord arterial pH < 7.10. The outcomes were compared between the groups defined according to cervical dilatation at diagnosis of labor dystocia and start of oxytocin infusion (≤ 5 cm, 6 -10 cm and fully dilated).

### Sample size estimation

The sample size calculation using Fischer´s exact test was based on rates of operative birth in TGCS group 1 in 2017 at the hospitals. With 183 women in each group (cervical dilatation ≤ 5 cm and 6–10 cm) a difference in rate of operative births (8 vs 17%) could be detected with a 0.05 level of significance at a power of 80%.

### Statistical analyses

All analyses were performed using SPSS Statistical package version 25.0 (IBM Corporation 1989, 2017). Categorical data is presented as number and per cent. Continuous data is presented as mean and one standard deviation (SD) or median and inter quartile range (IQR) if not normally distributed. Maternal characteristics and obstetric and neonatal outcomes were analyzed using a Chi^2^ test for categorical variables and when appropriate Fischer´s exact test, and a one-way ANOVA (Analysis of Variance) for continuous variables. A p-value of < 0.05 was considered statistically significant. Binary logistic regression was performed to calculate odds ratios (ORs), adjusted odds ratios (aORs) and 95% confidence intervals (95% CIs) for primary and secondary outcomes. The reference group was set at 6–10 cm according to the definition of active labor by ACOG [6]. In the binary logistic regression, CS and instrumental vaginal birth were merged into the outcome operative birth since there was no CS in the fully dilated group. The results were adjusted for maternal age at birth, BMI in early pregnancy and risk classification on admission to the labor ward, using a binary logistic regression model.

## Results

A total number of 588 women were included in the study. The women eligible for the study and the women who were excluded are presented in Fig. 1. A total number of 242 (41%) women were classified as low risk on admission to the labor ward and 346 (59%) as medium risk. None of the women were risk-classified as a high risk.

In the study population 34.5% had a diagnosis of labor dystocia and oxytocin infusion initiated at ≤ 5 cm cervix dilatation, 31.1% at 6–10 cm of cervical dilatation, and 34.3% at fully dilated cervix. The three groups were similar in terms of maternal characteristics, apart from a statistically significant difference in height, BMI in early pregnancy and risk classification on admission to the labor ward (Table 1). No women had pre-pregnancy hypertension, renal disease, preeclampsia, diabetes mellitus or heart disease.

**Table 1:** Maternal and obstetric characteristics of the study population

Cervix dilatation at diagnosis of dystocia and start of oxytocin infusion	≤ 5 cm *n* = 203	6–10 cm *n* = 183	Fully dilated *n* = 202	*P*-value
Age (years) mean [SD]	28.5 [4.8]	28.9 [4.3]	29.4 [4.3]	0.177
Smoking during pregnancy n (%)	5 (3)	5 (3)	1 (0.5)	0.202
Height (cm) mean [SD]	164.8 [6.0]	166.2 [6.0]	167.6 [6.1]	< 0.001
BMI (kg/m^2^) mean [SD]	25.7 [5.1]	25.8 [5.2]	24.5 [4.1]	0.013
Asthma/lung disease n (%)	21 (11)	8 (4)	18 (9)	0.075
Gestational age in days mean [SD]	283 [7.1]	282 [7.4]	281 [7.0]	0.14
Risk assessment on admission n (%)				< 0.001
Low risk	59 (29)	78 (43)	105 (52)	
Medium risk	144 (71)	105 (57)	97 (48)	
High risk	0	0	0	
Active phase of first stage of labor in minutes, median [IQR]	736 [515–958]	790 [619–968]	604 [451–764]	< 0.001
Second stage of labor in minutes, median [IQR]	36 [21–49]	31,5 [21–50]	37 [24–57]	0.114

Data is presented as mean and [standard deviation] or median and [inter quartile range] for continuous variables and number and (percent) for categorical variables. Percent was calculated within dilatation groups. *p* < 0.05 was considered statistically significant

*BMI* Body mass index, *IQR* Inter quartile range

The primary outcome, mode of birth in relation to cervical dilatation at diagnosis of dystocia and initiation of oxytocin infusion, is presented in Tables 2 and 3.

**Table 2:** Outcomes according to cervix dilatation groups at diagnosis of dystocia and start of oxytocin infusion

Cervix dilatation at diagnosis of dystocia and start of oxytocin infusion	≤ 5 cm *n* = 203	6–10 cm *n* = 183	Fully dilated *n* = 202	*P*-value
Spontaneous vaginal birth n (%)	149 (73.4)	144 (78.7)	179 (88.6)	< 0.001
Instrumental vaginal birth n (%)	29 (14.3)	27 (14.8)	23 (11.4)	0.569
Cesarean section n (%)	25 (12.3)	12 (6.6)	0 (0.0)	< 0.001
Need of epidural anesthesia n (%)	178 (88)	162 (89)	142 (70)	< 0.001
OASI grade III + IV n (%)	13 (6)	10 (5)	11 (5)	0.89
PPH (> 1000 mL) n (%)	14 (7)	10 (6)	14 (7)	0.80
Apgar < 7 at 5 min n (%)	8 (4)	4 (2)	1 (0.5)	0.06
Umbilical cord arterial pH < 7.10 n (%)	19 (12.2)	14 (10.3)	15 (9.7)	0.76
Negative birth experience (VAS 1–4) n (%)	34 (20.4)	14 (9.0)	19 (10.9)	0.006

Data is presented as number and percent. Percent was calculated within dilatation groups. *p* < 0.05 was considered statistically significant

*OASI* Obstetric Anal Sphincter injury, *PPH* Postpartum hemorrhage, *VAS *Visual Analog Scale

**Table 3:** Crude and adjusted odds ratios for obstetric and neonatal outcomes

	Crude OR (95% CI)	Adjusted OR^*^ (95% CI)
**Primary outcome**		
Operative birth		
≤ 5 cm	1.34 (0.84–2.14)	1.28 (0.78–2.08)
Fully dilated	0.47 (0.27–0.83)	0.48 (0.27–0.85)
*Secondary outcome*		
Need of epidural		
≤ 5 cm	0.92 (0.5–1.71)	0.91 (0.48–1.73)
Fully dilated	0.31 (0.18–0.53)	0.28 (0.16–0.50)
OASI grade III + IV		
≤ 5 cm	1.18 (0.51–2.77)	1.18 (0.50–2.81)
Fully dilated	0.10 (0.41–2.40)	1.07 (0.44–2.60)
PPH (> 1000 ml)		
≤ 5 cm	1.28 (0.56–2.96)	1.39 (0.60–3.28)
Fully dilated	1.29 (0.56–2.98)	1.36 (0.58–3.17)
Apgar score < 7 at 5 min		
≤ 5 cm	1.86 (0.55–6.27)	1.57 (0.45–5.46)
Fully dilated	0.23 (0.03–2.01)	0.22 (0.02–2.00)
Negative birth experience VAS 1–4		
≤ 5 cm	2.58 (1.32-5.01)	2.61 (1.30-5.29)
Fully dilated	1.24 (0.60-2.55)	1.39 (0.65-2.97)

Cervical dilatation at diagnosis of dystocia and start of oxytocin augmentation and the risk of operative birth (instrumental vaginal birth or cesarean section), adverse obstetric and neonatal outcomes and risk of negative birthing experience. Cervical dilatation of 6–10 cm at diagnosis of dystocia and start of oxytocin augmentation was set as reference

*OR* Odds Ratio, *OASI* obstetric anal sphincter injury, *VAS* Visual Analog Scale, *PPH* postpartum hemorrhage

^*^Adjusted for maternal age at birth, BMI in early pregnancy and risk assessment at admission to the labor ward

### Analog scale

The overall CS rate in the study population was 6.3%, 13.4% had an instrumental vaginal birth and 80.4% a spontaneous vaginal birth. The CS rate differed significantly between the women with a diagnosis of dystocia and start of oxytocin infusion at cervical dilatation ≤ 5 cm (12.3%) compared to the women with a cervical dilatation of 6–10 cm (6.6%) and the fully dilated group (none) (*p* < 0.001) (Table 2). Concurrently, there was a significant difference between the groups in women having a spontaneous vaginal birth with 73.4% in the ≤ 5 cm group, 78.7% in the 6–10 cm group and 88.6% in the fully dilated group. All instrumental vaginal births (*n* = 77) were vacuum extractions. The occurrence of instrumental birth did not differ significantly between the groups (14.3%, 14.8% and 11.4%) (Table 2). The corresponding outcome rates in the three dilatation groups (≤ 5 cm, 6-10 cm, fully dilated) for low-risk women (*n* = 242) were as follows, spontaneous vaginal birth 78.0%, 83.3%, 88.6%, and CS 6.8%, 2.6%, 0%.

Women diagnosed with dystocia and initiated oxytocin infusion when fully dilated had a decreased risk of operative birth (CS or instrumental vaginal birth) compared with women in the 6–10 cm cervical dilatation group (aOR 0.48 95% CI 0.27–0.85) even after adjusting for maternal age at birth, BMI in early pregnancy and risk classification on admission to the labor ward (Table 3). The secondary outcomes in relation to cervical dilatation at diagnosis of dystocia and initiation of oxytocin infusion are shown in Tables 2 and 3. The use of epidural anesthesia and negative birth experience (VAS 1–4) differed significantly between the three cervical dilatation groups (Table 2). Women with a diagnosis of dystocia and start of oxytocin infusion at ≤ 5 cm of dilatation, had an increased risk of reporting a negative birth experience (VAS 1–4), compared to women in the 6–10 cm group (aOR 2.61 (1.30-5.29)) (Table 3). Women in the fully dilated group had a decreased risk for having epidural anesthesia compared with women in the 6–10 cm group (aOR 0.28 95% CI 0.16–0.50) (Table 3).

## Discussion

This cohort study, including 588 women in the TGCS group 1, showed significant differences between the three cervical dilatation groups (≤ 5 cm, 6–10 cm, fully dilated) in rates of spontaneous vaginal births and CS, but no difference in rates of instrumental birth. The risk of operative birth (cesarean and vacuum extraction) was significantly lower in the fully dilated group compared with the 6–10 cm group, but no increased risk could be shown in the ≤ 5 cm group in comparison to the 6–10 cm group. Furthermore, women with a diagnosis of dystocia and start of oxytocin infusion at ≤ 5 cm had an increased risk of a negative birth experience.

These results are in line with a study by Häggsgård et al. who compared mode of birth among women in the TGCS group 1 according to the degree of cervical dilation when initiating labor augmentation with oxytocin. They found, in 464 women, that the more dilated the cervix was when initiating oxytocin augmentation, the higher the likelihood of a vaginal birth, and concluded that women who had oxytocin infusion initiated at ≤ 4 cm cervical dilatation had the highest risk of CS (13.6%) [21]. In this context it is also of interest to look at studies comparing mode of birth outcome according to cervical dilatation degree when active labor starts. Results from the present study are in line with results from a French study, where the definition of active labor was changed from 4 to 6 cm cervical dilatation, in which women who were diagnosed with labor dystocia and had oxytocin infusion was initiated before 7 cm had an increased risk of CS [18]. On the other hand, when the Norwegian LaPS trial cluster-randomized women in the TGCS group 1 to active labor defined as either 4 or 6 cm of cervical dilatation, they found no difference in mode of delivery. However, the total CS rate in both groups decreased during the trial, from 9–10% to 6% [19]. One reason for the current study’s incoherence with the LaPS-trial might be due to the differences in study design. More studies are needed to evaluate both mode of birth and neonatal outcomes in women with and without interventions due to labor dystocia at low cervical dilatation degrees.

In the present study population 34.5% were diagnosed with dystocia and received oxytocin infusion when the cervix dilatation was ≤ 5 cm and were thus in the latent phase of labor according to the ACOG definition [6] but not according to the Swedish criteria of active labor [12]. We found that in the ≤ 5 cm group the CS rate was twice as high as in in the 6–10 cm group. With the proposed definition of start of active labor at a cervical dilatation of 6 cm [6, 11], oxytocin infusion before that would be classified as induction of labor in the latent phase of labor, instead of spontaneous onset and labor augmentation due to labor dystocia. It is well described that the rates of CS increase when labor is induced compared to spontaneous onset of labor [22], and the increased rate of CS in the ≤ 5 cm group in the present study might have been because these women were still in the latent phase of first stage of labor when diagnosed with labor dystocia and oxytocin infusion was initiated. As women enter the active stage of labor the cervical collagen structure transforms to become softer and more prone to dilatation [23]. If the contractions are reinforced with oxytocin infusion before the cervix has changed its structure and thus remains firm, it might not be able to dilate and thereby there is no progress of labor.

The current study found that 20% of the women that were diagnosed with labor dystocia and had oxytocin augmentation initiated at ≤ 5 cm of dilatation had a negative birthing experience measured by VAS, compared to one out of five women with start of augmentation at 6–10 cm cervical dilatation. A negative birthing experience has in earlier studies been associated with a long duration of labor and CS [1, 24, 25], oxytocin augmentation during the first stage of labor [24, 26], instrumental birth and PPH [24]. Satisfaction with childbirth experience is a measure of quality and should be a significant endpoint according to the WHO, alongside the outcome of healthy mother and healthy baby. WHO further states that the increased medicalization of normal childbirth deprives women of their own birthing capabilities and contributes to a higher risk of a negative childbirth experience [8]. The individual parts of the cascade of interventions in women with diagnosed labor dystocia have not been evaluated in relation to the women’s birth experience in the current study. Factors of importance could be that the use of oxytocin infusion not only restricts women’s mobility during labor and birth due to the increased need of continuous CTG, but also increase the risk of more discomfort, pain and need for epidural analgesia [27].

### Strengths and limitations

This study has certain strengths and limitations. One strength is the large study population of term nulliparous women with spontaneous onset of labor and oxytocin augmentation initiated during labor, enabling evaluation of outcomes in three cervical dilatation groups with cut-offs customized to the latest definitions on start of active labor. Another strength is the cohort design, where all women during a specified period of time were included in the study, which minimized the risk of selection bias. The detailed prospectively collected data on baseline evaluation of maternal comorbidity and socioeconomic factors, enabled adjustment for possible confounding factors. Another strength is the availability of manually extracted risk assessments, which made it possible to adjust outcomes for the woman´s individual risk on admission to the labor ward.

The retrospective design of a study is always a limitation as the researcher has no control over the data entered into the electronic medical records. Also, additional data that would have been valuable in the analysis (e.g. cervix dilatation on admission or time from start of oxytocin infusion to birth) was not available. The choice of confounding factors was based on previous similar research and clinical experience, but there might be unknown confounding factors that could have biased our results. Another drawback arose when it became apparent that no CS were performed in the group which had oxytocin initiated when cervix was fully dilated. A composite outcome of CS and instrumental birth was therefore created and named “operative birth”, enabling the data to be further analyzed using binary logistic regression. The context in which this study was performed has a long tradition of high use of oxytocin but also high frequencies of spontaneous vaginal births, a fact that might reduce the generalizability to other populations with higher incidence of CS.

## Conclusion

This study on nulliparous women with spontaneous onset of labor and labor dystocia, performed in a low CS setting, showed a significant difference in mode of birth rates among the three cervical dilatation groups. The fact that the highest rate of CS occurred when labor dystocia was diagnosed and oxytocin was initiated before ≤ 5 cm of cervical dilatation might indicate that oxytocin augmentation before 6 cm could be contra productive in preventing CS. Additionally, the higher risk for a negative birth satisfaction among the women in the ≤ 5 cm of cervical dilatation group, calls for caution when considering augmenting labor at ≤ 5 cm of cervical dilatation. The results from the present study support the shift toward a definition of active labor at a higher cervical dilatation degree, minimizing interventions in early stages of labor, and thus potentially increasing both the number of spontaneous vaginal births and women´s satisfaction with childbirth. These results should be considered when designing new recommendations on labor care.

### Supplementary Information

**Additional file 1: Appendix 1. **Risk assessment on admission to the labor ward. 

## Data Availability

The datasets generated and/or analyzed during the current study are not publicly available due to restrictions in Swedish law (Offentlighets- och sekretesslag (SFS 2009:400)/Public Access to Information and Secrecy Act (SFS 2009:400)) but are available from the corresponding author on reasonable request.

## Abbreviations

ACOG: American College of Obstetricians and Gynecologists
ANOVA: Analysis of Variance
aORs: Adjusted odds ratios
BMI: Body Mass Index (kg/m^2^)
CIs: Confidence intervals
CS: Cesarean section
CTG: Cardiotocography
IQR: Inter Quartile Range
OASI: Obstetric anal sphincter injury
ORs: Odds ratios
PPH: Postpartum hemorrhage
TGCS: Ten Group Classification System
WHO: World Health Organization

## References

[1] Selin L, Wallin G, Berg M (2008). Dystocia in labour–risk factors, management and outcome: a retrospective observational study in a Swedish setting. Acta Obstet Gynecol Scand.

[2] Cheng YW, Shaffer BL, Bryant AS, Caughey AB (2010). Length of the first stage of labor and associated perinatal outcomes in nulliparous women. Obstet Gynecol.

[3] Nyfløt LT, Stray-Pedersen B, Forsén L, Vangen S (2017). Duration of labor and the risk of severe postpartum hemorrhage: a case-control study. PLoS ONE.

[4] Selin L, Almström E, Wallin G, Berg M (2009). Use and abuse of oxytocin for augmentation of labor. Acta Obstet Gynecol Scand.

[5] Socialstyrelsen. Indikation för värkstimulering med oxytocin under aktiv förlossning (Indication for augmentation with oxytocin during active labor). 2011 2011 08/01 Report No 2011 18

[6] Caughey AB, Cahill AG, Guise J-M, Rouse DJ, American College of Obstetricians and Gynecologists. Safe prevention of the primary cesarean delivery. Am J Obstet Gynecol. 2014;210(3):179–93. 10.1016/j.ajog.2014.01.026 24565430

[7] World Health Organization. WHO labour care guide: user’s manual. Geneva; 2020.

[8] World Health Organization. WHO recommendations: intrapartum care for a positive childbirth experience. Geneva; 2018. 30070803

[9] Friedman EA (1954). The graphic analysis of labor. Am J Obstet Gynecol.

[10] Lundborg L, Åberg K, Sandström A, Discacciati A, Tilden EL, Stephansson O (2020). First stage progression in women with spontaneous onset of labor: A large population-based cohort study. PLoS ONE.

[11] Zhang J, Landy HJ, Branch DW, Burkman R, Haberman S, Gregory KD (2010). Contemporary patterns of spontaneous labor with normal neonatal outcomes. Obstet Gynecol.

[12] Perinatal ARG och svenska Barnmorskeförbundet. Definition av etablerat förlossningsarbete (Definition of active labor). 2015.

[13] World Health Organization. Robson Classification: Implementation Manual. Geneva; 2017.

[14] Robson M (2015). The Ten Group Classification System (TGCS)–a common starting point for more detailed analysis. BJOG.

[15] Boerma T, Ronsmans C, Melesse DY, Barros AJ, Barros FC, Juan L (2018). Global epidemiology of use of and disparities in caesarean sections. Lancet.

[16] Blomberg M (2016). Avoiding the first cesarean section–results of structured organizational and cultural changes. Acta Obstet Gynecol Scand.

[17] Caughey AB, editor Is Zhang the new Friedman: How should we evaluate the first stage of labor? Semin Perinatol; 2020: Elsevier. 10.1016/j.semperi.2019.151215 32067749

[18] Thuillier C, Roy S, Peyronnet V, Quibel T, Nlandu A, Rozenberg P (2018). Impact of recommended changes in labor management for prevention of the primary cesarean delivery. Am J Obstet Gynecol.

[19] Bernitz S, Dalbye R, Zhang J, Eggebø TM, Frøslie KF, Olsen IC (2019). The frequency of intrapartum caesarean section use with the WHO partograph versus Zhang's guideline in the Labour Progression Study (LaPS): a multicentre, cluster-randomised controlled trial. Lancet.

[20] Graviditetsregistret (The Swedish Pregnancy Registry) [Internet]. 2020 [cited 16 Dec 2020]. Available from: https://www.medscinet.com/gr/default.aspx.

[21] Häggsgård C, Persson EK (2020). Management of oxytocin for labour augmentation in relation to mode of birth in Robson group 1. Midwifery.

[22] Grivell RM, Reilly AJ, Oakley H, Chan A, Dodd JM (2012). Maternal and neonatal outcomes following induction of labor: a cohort study. Acta Obstet Gynecol Scand.

[23] Uldbjerg N, Ekman G, Malmström A, Olsson K, Ulmsten U (1983). Ripening of the human uterine cervix related to changes in collagen, glycosaminoglycans, and collagenolytic activity. Am J Obstet Gynecol.

[24] Falk M, Nelson M, Blomberg M (2019). The impact of obstetric interventions and complications on women’s satisfaction with childbirth a population based cohort study including 16,000 women. BMC Pregnancy Childbirth.

[25] Nystedt A, Hildingsson I (2014). Diverse definitions of prolonged labour and its consequences with sometimes subsequent inappropriate treatment. BMC Pregnancy Childbirth.

[26] Johansson C, Finnbogadóttir H (2019). First-time mothers’ satisfaction with their birth experience–a cross-sectional study. Midwifery.

[27] Wei S-Q, Luo Z-C, Xu H, Fraser WD (2009). The effect of early oxytocin augmentation in labor: a meta-analysis. Obstet Gynecol.

