# Correction: Labor dystocia and oxytocin augmentation before or after six centimeters cervical dilatation, in nulliparous women with spontaneous labor, in relation to mode of birth

**DOI:** 10.1186/s12884-024-06292-7

**Published:** 2024-02-02

**Authors:** Cecilia Brüggemann, Sara Carlhäll, Hanna Grundström, Marie Blomberg

**Affiliations:** 1 Department of Obstetrics and Gynecology in Linköping, Department of Biomedical and Clinical Sciences, Linköping University, Linköping, Sweden; 2 Department of Obstetrics and Gynecology in Norrköping, Department of Biomedical and Clinical Sciences, Linköping University, Linköping, Sweden

**Correction: BMC Pregnancy Childbirth 22, 408 (2022)**

10.1186/s12884-022-04710-2

Following publication of the original article [1], the authors would like to correct the calculations of negative birth experience measured by VAS score from 1 to 10. Missing values in the database were marked with 0 (zero). Unfortunately, when performing calculations of the VAS 1–4 group, which was the definition of a negative birth experience, missing values (marked with 0) were added to the VAS 1–4 group. When excluding 0 (0 = missing value on VAS) the overall results presented in the article did not change, hence the associations became stronger, and the prevalence of negative birth experience decreased.

The original article [1] has been corrected.
