# Supplementary material for: Labor dystocia and oxytocin augmentation before or after six centimeters cervical dilatation, in nulliparous women with spontaneous labor, in relation to mode of birth
Source: BMC Pregnancy Childbirth. 2022 May 13;22:408. doi: 10.1186/s12884-022-04710-2 (PMC9107162; doi:10.1186/s12884-022-04710-2)
Supplement: Supplementary file 1 — Additional file 1: Appendix 1. Risk assessment on admission to the labor ward. [file 12884_2022_4710_MOESM1_ESM.docx]

**APPENDIX 1** Risk assessment on admission to the labor ward.

**Low risk**

- Uncomplicated pregnancy
- Gestational week 37+0 - 41+6
- Single gestation
- Spontaneous onset of labor
- Rupture of membranes with no meconium-stained amniotic fluid with spontaneous contractions
- Normal CTG trace according to classification system
- Blood pressure < 140/90
- No (severe) medical history

**Medium risk**

Criteria for low risk not fulfilled

*Complicated obstetrical history*

- IUFD (intra uterine fetal death)
- Preeclampsia
- Placental retention
- Postpartum hemorrhage >2000ml
- Cesarean section
- OASI (obstetric anal sphincter injury)
- Shoulder dystocia

*Complicated pregnancy*

- Fear of childbirth
- Blood infections (HIV; hepatitis etc.)
- BMI > 30 in early pregnancy
- Drug or alcohol abuse
- Twin-pregnancy
- GBS-bacteria in urine
- Impending premature birth
- Immunization
- Induction of labor
- Placenta praevia
- Poly/oligohydramnios
- Preeclampsia
- Ongoing medical history (diabetes, epilepsy, severe psychiatric disease)
- Breech presentation
- Growth retardation
- ≥ 42+0 gestational weeks
- Older nulliparous women (>40 years at consummation of pregnancy)
- Multipara > 4

*Complications during labor*

- Non-reassuring CTG trace
- Maternal fever
- Malpresentation of fetus
- Hemoglobin <90g/L
- IUFD or late termination of pregnancy
- Communication difficulties
- Heavily meconium-stained amniotic fluid
- Long labor
- Rupture of membranes > 18 h
- Vaginal bleeding (not bloody show)

**High risk**

Seriously ill patients, for example severe preeclampsia or HELLP (hemolysis, elevated liver enzymes and low platelet count) or current heart condition.
